# The Effects of High-Intensity Interval Training vs. Moderate-Intensity Continuous Training on Heart Rate Variability in Physically Inactive Adults

**DOI:** 10.3390/ijerph15071508

**Published:** 2018-07-17

**Authors:** Abdullah Alansare, Ken Alford, Sukho Lee, Tommie Church, Hyun Chul Jung

**Affiliations:** 1 Department of Kinesiology, College of Health Sciences, University of Louisiana at Monroe, 700 University Avenue Brown Hall, Monroe, LA 71209, USA; aalansare@ksu.edu.sa (A.A.); alford@ulm.edu (K.A.); church@ulm.edu (T.C.); 2 Department of Exercise Physiology, College of Sport Sciences and Physical Activity, King Saud University, King Khalid Rd, Riyadh 11543, Saudi Arabia; 3 Department of Counseling, Health, and Kinesiology, College of Education and Human Development Texas A&M University-San Antonio, One University Way, San Antonio, Texas, TX 78224, USA; slee@tamusa.edu

**Keywords:** cardiac auto-regulation, frequency domain, time domain, blood pressure, exercise

## Abstract

Physically inactive adults are prevalent worldwide. This study compared the effects of short-term high-intensity interval training (HIIT) versus moderate-intensity continuous training (MICT) on heart rate variability (HRV) in physically inactive adults as a preliminary study. Thirteen physically inactive male adults (27.5 ± 3.80 years) were randomly assigned to HIIT (N = 7) or MICT (N = 6). The HIIT program consisted of 20 min of interval training with cycling to rest ratio of 10/50 s at ≥90% HR_peak_, while the MICT program consisted of 40 min of continuous cycling at 60–75% HR_peak_. Both groups completed eight sessions of training within two weeks. Time and frequency domains of HRV were measured for 20 min with Actiwave-Cardio monitor (CamNtech, UK). The number of R-R interval and inter-beat interval (IBI) were significantly improved (*p* < 0.05) in both HIIT and MICT programs following eight sessions of training. A significant interaction effect for group by time was found in the lnLF/HF ratio (*p* < 0.05) where it was only improved in the HIIT group from pre- to post-test. The HIIT program is superior to MICT in improving HRV in physically inactive adults. The HIIT program can be applied as a time-efficient program for improving cardiac-autoregulation.

## 1. Introduction

Physical inactivity, which is an independent risk factor for several chronic diseases, is globally prevalent [1]. One out of five adults are physically inactive worldwide [2], and more than 247 million adults are currently physically inactive in the United States [3]. Being physically inactive increases risk factors for mortality including cardiovascular diseases, cancer, and diabetes [4,5]. Shifting to an active lifestyle by being involved in physical activity programs would prevent such pathologies and improve overall health [6].

The World Health Organization (WHO) generally recommends adults to engage in moderate-intensity of physical activity for 150 min/week to maintain their cardiovascular health [1]. Although many health organizations including WHO emphasize the importance of physical activity programs, the number of inactive adults remains high [2,3]. The lack of time seems to be the main contributor to the prevalence of physical inactivity among adults [7]. Therefore, a modified exercise protocol that is time efficient and has similar or better effects on cardiovascular health may increase the number of participants in physical activity programs.

High-intensity interval training (HIIT), which consists of repeated exercises at high-intensity separated with short recovery periods, has received more attention by exercise physiologists in last two decades. HIIT has been used as a time-efficient program to improve physical fitness, physiological functions, and cardiovascular disease risk factors [8,9]. Furthermore, HIIT was chosen as the most enjoyable physical activity program compared to moderate to vigorous continuous training [10].

The assessment of heart rate variability (HRV) has been widely applied as a non-invasive method that measures the time between heartbeats and electrical cardiac activity to evaluate the cardiovascular health. HRV is linked to various chronic diseases including metabolic and cardiovascular diseases [11]. HRV can be evaluated by analyzing its two domains: time and frequency [12]. The time domain is used to calculate the number of heartbeats and the time intervals between beats at any point. For example, the inter-beat interval (IBI) represents the measurement of the time interval between heartbeats while the number of R-R interval (No. R-R interval) indicates the number of heartbeats within a certain period, usually within five minutes [11]. The frequency domain is often used to evaluate the cardiac-autonomic responses. This domain includes high frequency (HF), an indication of vagal activity (parasympathetic activity), low frequency (LF), which indicates both sympathetic and parasympathetic activity but more dominant by sympathetic activity, and LF/HF ratio that represents of sympathovagal tone [13].

Up to now, several studies have been existed to confirm the effectiveness of HIIT program on HRV in various populations such as cardiac or diabetic patients and inactive adults [9,14,15]. Short- and long-terms moderate-intensity continuous training (MICT) also showed HRV improvements in physically inactive adults [16,17]. However, it remains unclear how the HRV outcomes respond following HIIT and MICT programs in physically inactive adults. If HIIT program has similar or greater benefits on HRV compared to MICT, then it would become a favorable program for time-constrained physically inactive adults. Therefore, the present study was designed to compare the effects of repeated short-term high-intensity interval training (HIIT) and moderate-intensity continuous training (MICT) on heart rate variability (HRV) in physically inactive adults as a preliminary study. It was hypothesized that HIIT would be more efficient for improving HRV in physically inactive adults.

## 2. Materials and Methods

### 2.1. Experimental Design

While various health organizations state the importance of physical activity, the prevalence of physically inactive adults remains high. As time constraints plays a key role in this prevalence, time-efficient exercise protocols should be developed to increase the physical activity level. The present study compared the effects of two different exercise protocols (HIIT vs. MICT) on heart rate variability in physically inactive adults. This study was conducted in a randomized and controlled design. First, subjects’ height, body weight, body circumference and resting blood pressure were measured. Then, VO_2peak_ test was performed on a cycle ergometer to determine the exercise intensities. The eligible subjects were randomly allocated to HIIT or MICT group. All subjects performed eight sessions of cycling exercise over a two-week period. Heart rate and rate of perceived exertion (RPE) were recorded during training. Blood pressure and HRV were measured two times (pre- and post-tests) three days before and after the intervention to avoid the effect of the last training session. All subjects were instructed not to drink any caffeine-contained beverages 24–48 h prior the tests nor participate in other exercise programs. All measurements were performed in the morning between 8:00 a.m. to 9:00 a.m.

### 2.2. Participants

**Figure 1:**
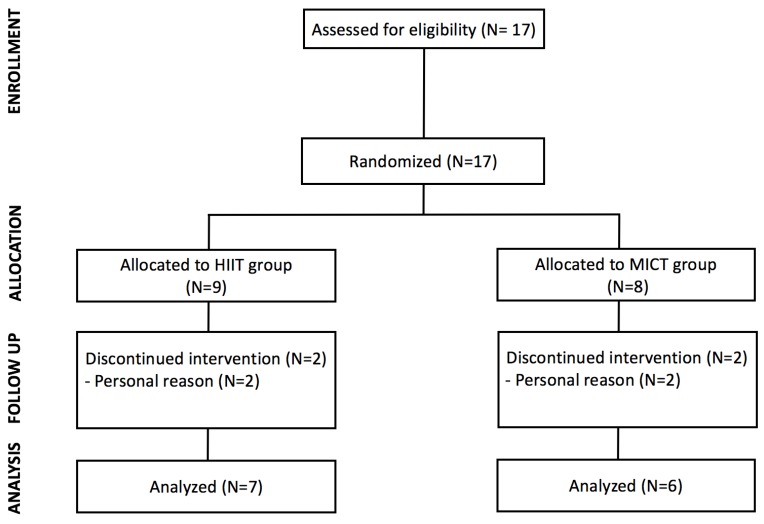
Study flowchart.

Initially, 17 male subjects who met the inclusion criteria, participated in this study. The inclusion criteria were: (a) to be male adults, aged between 20 to 40 years, (b) to be able to do cycling exercise, and (c) to be physically inactive for last six months. Physically inactive adults were defined as individuals who did not meet the physical activity recommendations of 150 min/week of moderate-intensity exercise [18]. Any subjects who had knee and/or lower back pain or any cardiovascular- related diseases were excluded. The eligible subjects were randomly assigned to either HIIT or MICT group. During the intervention period, four subjects dropped out due to personal reasons. Therefore, thirteen subjects (HIIT, N = 7, MICT, N = 6) completed the study (Figure 1). This study was approved by the institutional review board (IRB) of the University, and written consent form was obtained from each subject. The characteristics of subjects are presented in Table 1.

### 2.3. Measurements

#### 2.3.1. Blood Pressure

Systolic and diastolic blood pressures were measured with an electronic sphygmomanometer (Advantage, Model No-6021, American Diagnostic Corporation, Hauppauge, NY, USA). Prior the measurement of blood pressure, subjects sat on a chair for at least five minutes with back support, feet on the floor and arms supported at heart level to allow the blood pressure to be adjusted. The blood pressure was measured twice with one-minute interval between the measurements. Interclass coefficient (ICC) of systolic and diastolic blood pressure were 0.89 and 0.93 respectively. The average score of the two measures was recorded nearest to one mmHg.

#### 2.3.2. Peak Oxygen Uptake

A peak oxygen uptake test (VO_2peak_) was performed on a cycle ergometer with Metabolic cart (Quark CPET, Cosmed, Italy) to determine the exercise intensities. Subjects began cycling at a load of 50 watts, and 25 watts were gradually added every two minutes until the subjects were exhausted. All subjects were instructed to maintain their speed at 60 revolutions per minute (RPM) throughout the test, and verbal encouragements were given to all subjects. Heart rate was monitored every minute by chest-strap heart rate monitor (Polar T31^TM^ transmitter, Polar Electro, Kempele, Finland U), and RPE was obtained every two minutes. All subjects performed five minutes warm-up and cool-down at low-intensity load (50 watts). VO_2peak_ and HR_peak_ were determined when subjects met at least two of following conditions: (a) inability to keep up the pedaling at rate of 60 rpm for more than five seconds with verbal encouragement, (b) respiratory exchange ratio (RER) >1.15, (c) rating of perceived exertion (RPE) ≥19 (Borg 6–20 scale), or (d) volitional fatigue.

#### 2.3.3. Heart Rate Variability

Subjects were instructed not to drink any caffeine-contained products 24–48 h prior to HRV measurement. Actiwave Cardio monitor (CamNtech, Cambridge, UK), which traces the electrocardiogram (ECG), was used to measure the HRV variables. The device has been used for HRV study and the validation has been approved in a previous study [19,20]. Subjects visited the laboratory in the morning between 8:00 am to 9:00 am. Before performing any physical test, subjects were seated on a comfortable chair in a quiet room with light turned off. They were instructed to remain quiet for HRV measurement. Two ECG electrodes were adhered at subjects’ upper chest (5th intercostal space and 10 cm away on the left side at Lead four and Lead five). Sample rate and resolution of collected data was set at 1024 Hz with 10 bits. The resting HRV test was performed for 20 minutes with sitting position without any movements. The first five minutes of the 20 minutes were ignored to avoid the collecting of noisy data which might exist due to the change of body position (from standing to sitting position), and then the following clear five minutes were selected according to the standards of HRV analysis by Task Force of the European Society of Cardiology [12]. The data were analyzed by using a computer software program (Activewave Cardio analysis version 3.0.8, CamNtech, Cambridge, UK). Both time (i.e., number of R-R interval, IBI, root mean square of the successive differences; RMSSD) and frequency domains (LF, HF, LF/HF ratio) were analyzed. To ensure the accuracy of the entire measurements, the same investigator analyzed both pre- and post-tests.

### 2.4. Exercise Interventions

Cycling exercises were performed at the human performance laboratory. Subjects were randomly assigned to participate four times a week for two weeks in HIIT or MICT program. The training intensity and duration were modified from previous studies [10,21]. Cycle ergometers (Monark 828E, Monark, Sweden) were used in both programs. HIIT consisted of 20 min/session cycling at intensity of ≥90% of heart rate peak (HR_peak_). Each minute of the 20 min was separated to two phases: active and recovery phases (10/50 s respectively). During the active phase, subjects performed maximal cycling at speed of 100 RPM or higher and decreased to lower than 50 RPM during recovery phase. MICT consisted of 40 min continuous cycling at intensity of 60–75% of HR_peak_, and subjects performed cycling with maintaining their speed at 60 RPM. Subjects in both groups performed five minutes cycling warm-ups and five minutes cycling cool downs at a low-intensity (40% HR_peak_) during each visit. RPE and HR were recorded every five minutes during cycling.

### 2.5. Data Analysis

The analysis was performed by using SPSS for windows (version 25, SPSS Inc., Chicago, IL, USA). All data were expressed as means and standard deviations. A natural logarithmic transformation of LF and HF powers were performed to meet the assumptions of parametric statistical analysis. The statistical descriptive test was analyzed to examine the general characteristics of the subjects such as height, weight and VO_2peak_. The independent t-test was applied to compare the baseline between the groups to ensure no significant differences at the baseline. A repeated measure analysis of variable (ANOVA) was used to examine the interaction effect (group × time) for all variables. When significant interaction or main effects were detected, the independent and paired *t*-test were applied. A significant level was set at *p* < 0.05.

## 3. Results

### 3.1. Training Information

Both groups completed eight sessions of training over two weeks. Training load was significantly heavier in the HIIT program than the MICT program (*t* = 3.967, *p* < 0.05). Average HR and RPE were significantly higher in the HIIT program compared to the MICT program (*t* = 2.489, *t* = 4.048, *p* < 0.05 respectively). However, total duration of training was significantly longer in the MICT than the HIIT programs (*t* = 14.231, *p* < 0.05) (Table 2).

### 3.2. Changes in Blood Pressure

There was a significant main effect in systolic blood pressure (*p* < 0.05) where it was improved in both HIIT and MICT groups from pre- to post-tests. However, no significant interaction or main effects were observed in diastolic blood pressure (Table 3).

### 3.3. Changes in Heart Rate Variability

**Figure 2:**
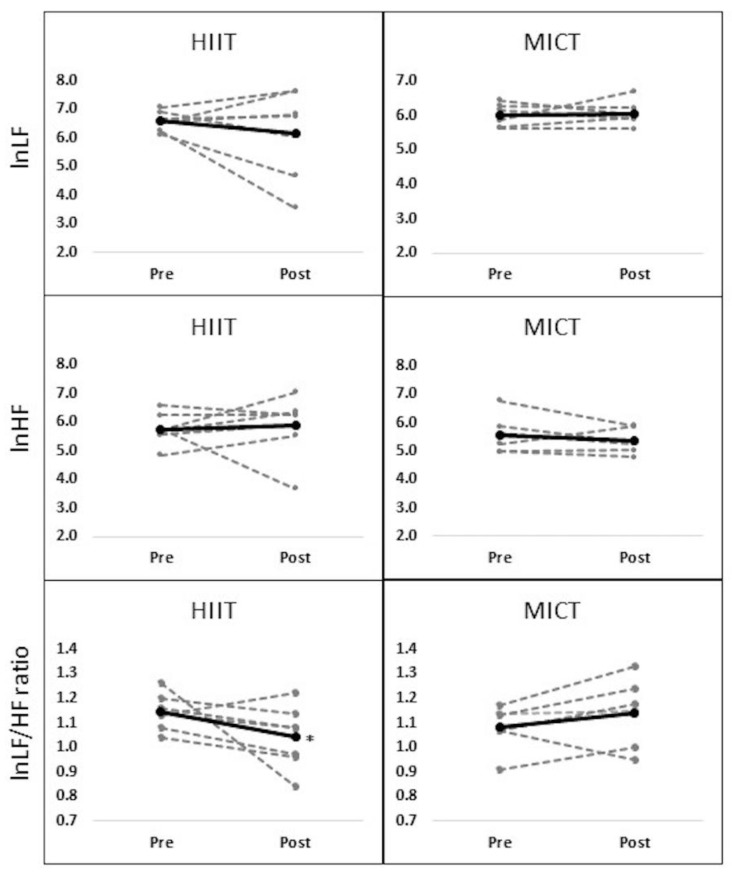
Changes in frequency domain variables from pre- to post-tests. Note: Significant interaction effect for group by time on lnLF/HF ratio was observed where HIIT group only decreased from pre- to post-tests (*p* < 0.05), Dash line indicates the individual score while solid line indicates the average score in the HIIT and MICT groups.

There were significant time effects on the number of R-R interval (*p* < 0.05) and IBI (*p* < 0.05) where they were improved in both groups from pre- to post-tests. However, no significant interaction or main effects were observed in RMSSD (Table 4). Regarding frequency domain, there was a significant interaction effect for group by time in lnLF/HF ratio (F = 4.875, *p* < 0.05) where it was improved in the HIIT group only following eight sessions of training (Figure 2). However, other variables including lnLF and lnHF did not significantly change over the time.

## 4. Discussion

This study compared the effects of short-term HIIT and MICT programs on HRV in physically inactive adults. The main findings were that both HIIT and MICT programs significantly improved the systolic blood pressure and time domains of HRV including the number of R-R interval and IBI. However, lnLF/HF ratio was only significantly improved in the HIIT group following eight sessions of training.

Mounting evidence demonstrates that high blood pressure is associated with mortality and cardiovascular diseases [22]. Participating in physical activity programs have been recommended as an effective non-pharmacological approach to improve blood pressure [4]. In the same regards, the present study showed that two weeks of HIIT and MICT programs decreased systolic blood pressure in physically inactive adults. A previous study reported two weeks of HIIT (4–6 sets of Wingate exercise with 4–5 mins recovery between trials) improved systolic blood pressure in sedentary overweight and obese adults [23]. Even a single bout of HIIT (6 × 1 min at 98% maximum wattage with 4 min interval rests) decreased both peripheral and central blood pressure in healthy adults [24]. The short-term MICT program also improved blood pressure in adults. As an example, four weeks MICT at 65% of VO_2peak_ were sufficient to decrease blood pressure in normotensive and hypertensive patients [25]. Several physiological explanations such as improved vascular functions and diameter have been proposed to elucidate blood pressure improvements following physical activity programs such as HIIT and MICT programs [26]. Although the vascular diameter was not directly measured in the current study, previous studies found that both a single bout or repeated HIIT and MICT exercises beneficially influenced the vascular diameter (i.e., flow-mediated dilation) that results in systolic blood pressure improvement [27,28]. Accordingly, it is carefully assumed that vascular adaptation to HIIT and MICT may be associated with systolic blood pressure improvement in the current study, but our assumption cannot be confirmed unless the vascular diameter is measured.

In the present study, the time domain variables (the number of R-R interval and IBI) of HRV were improved following eight sessions of HIIT and MICT programs in physically inactive adults. Similar improvements were found in previous studies. For example, a study showed that twelve weeks of HIIT at 85-95% intensity of their maximal heart rate improved time domains of HRV in physically inactive adults [9]. A similar result after the MICT program has been observed in a previous study. A previous study showed that six months of MICT at 60–85% HR_max_ improved R-R intervals in diabetic adults [29]. Substantially, a decrease in resting heart rate (the number of R-R interval) has been identified as one of the early cardiac adaptations in response to repeated exercises [26]. This adaptation could be explained by alternative improvements of intrinsic heart rate (SA node) and vagal activity (parasympathetic activity) [26]. Interestingly, resting RMSSD, an indication of the parasympathetic activity, did not significantly change following both training programs despite the resting heart rate improvements (R-R interval, IBI). A previous study reported similar findings that the number of R-R interval was improved without RMSSD changing following HIIT and MICT programs [9]. As opposed to these findings, several studies reported that RMSSD was improved along with other time domain variables (i.e., R-R interval, IBI) following HIIT or MICT programs [15,30]. Smith & Fernhall pointed out that reduction in resting heart rate could be achieved by lowering intrinsic heart rate (lowering SA node activity) even without autonomic nervous activity improvements (sympathetic and parasympathetic activities) [26]. Therefore, it was assumed that the resting heart rate changes (R-R interval, IBI) might be associated with SA node improvement in the present study.

Regarding the frequency domain, lnLF/HF ratio, which refers to sympathovagal activity, was improved only in the HIIT group without significant changes in lnLF nor lnHF. It has been demonstrated that a single session or repeated HIIT programs improved lnLF/HF ratio in adults with cardiovascular disease [14,15], while MICT programs did not show sympathovagal improvement [31,32]. It was assumed that the intensity of exercise might play an essential role in modulating HRV variables such as frequency domains [33]. In contrast to low and moderate intensities of exercises, high and intensive exercises elevate the release of epinephrine and norepinephrine hormones, which are associated with sympathetic activity [34]. A greater sympathetic activity during HIIT may facilitate the improvement of cardiac function (i.e., auto-regulation, cardiac output) within short-period time compared to MICT. Accordingly, when comparing the results of the current study with previous studies, it was noticed that exercise intensity of ≥80% resulted in frequency domain’s improvements [14,31,35]; however, intensity of ≤80% did not influence the frequency domain [30,32].

Although the current study did not show any significant change in lnHF, an index of parasympathetic activity, the percent changes in this variable was positive in the HIIT group (+2.3%) while lnHF decreased in the MICT group (−6.3%). The change of sympathovagal activity (lnLF/HF ratio) in the HIIT group might be associated with parasympathetic the improvement (lnHF). While a parasympathetic activity (HF) improved following a HIIT program [14], this positive change was not found following MICT programs [30,32]. It is assumed that short-term HIIT program may provide a benefit on HRV variables, mostly in parasympathetic activities (i.e., HF, LF/HF ratio).

There are some limitations that should be considered when interpreting the results of this study. The present study was systematically designed; however, the number of enrolled subjects was small (*n* = 13). It is believed that the adequate number of subjects are required in clinical interventions to provide precise conclusions [36]. Even though the present study showed significant HRV changes, future replicated studies with large sample size are needed to get precise results and conclusions about the effects of short-term HIIT and MICT on HRV in physically inactive adults. The single-gender subject might be another limitation in this study. Since there was a gender difference on cardiovascular adaptation in response to exercise [37,38], the recruited subjects in this study were only inactive male adults. Thus, the effects of short-term HIIT compared to MICT on HRV in inactive women might differ from the present findings. Another limitation is that the subjects were only young adults (20–40 years old). It is well known that the cardiovascular responses to various stimuli differ from young to elderly individuals [39]. Thus, it should be considered that the results might differ when compared with elderly or adolescents.

## 5. Conclusions

The present study revealed that eight sessions of HIIT and MICT programs improve systolic blood pressure and time domains of HRV including the number of R-R intervals and IBI. However, sympathovagal (lnLF/HF ratio) activity was only improved following eight sessions of HIIT. This study suggests that the HIIT program is superior to MICT in improving HRV in physically inactive adults. However, future studies with large sample sizes are needed to confirm the effects of HIIT on HRV in physically inactive adults.

## Figures and Tables

**Table 1:** Characteristics of subjects.

Variables	Mean ± SD
Age (yrs)	27.5 ± 3.80
Height (cm)	173.9 ± 8.56
Body weight (kg)	90.2 ± 19.24
Body mass index (kg/m^2^)	29.7 ± 5.89
Waist-hip ratio	0.8 ± 0.03
Systolic BP (mmHg)	123.5 ± 4.60
Diastolic BP (mmHg)	75.4 ± 5.67
HR_rest_ (beat/min)	67.5 ± 10.67
HR_peak_ (beat/min)	174.6 ± 20.72
VO_2peak_ (ml/kg/min)	29.8 ± 6.40

BP: blood pressure; HR_rest_: resting heart rate, HR_peak_: peak heart rate, VO_2peak_: peak oxygen uptake.

**Table 2:** Descriptive training data between high-intensity interval training (HIIT) and moderate-intensity continuous training (MICT) groups.

	HIIT	MICT
Completed session (n)	8	8
Exercise load (kp)	5.0 ± 2.33 *	1.2 ± 0.21
Average heart rate (beat/min)	159.8 ± 12.33 *	139.3 ± 17.35
Average RPE (score)	15.6 ± 1.30 *	11.0 ± 2.70
Total exercise duration (min)	159.4 ± 1.15 *	305.4 ± 27.29

Values are expressed mean and standard deviations. * *p* < 0.05, indicates a significant difference between HIIT and MICT.

**Table 3:** Changes in blood pressure from pre-to post-tests.

		PRE	POST	Group	Time	G × T
SYS BP (mmHg)	HIIT	123.7 ± 4.57	118.0 ± 3.94 ^+^	0.726	14.495 *	0.128
	MICT	122.3 ± 5.11	115.4 ± 6.34 ^+^			
DIA BP (mmHg)	HIIT	73.6 ± 2.40	70.9 ± 6.6	0.651	2.148	0.007
	MICT	75.5 ± 6.49	72.5 ± 3.59			

SYS BP: Systolic blood pressure, DIA BP: diastolic blood pressure. Values are expressed mean and standard deviations. * *p* < 0.05, indicates significant time or interaction effect, ^+^ *p* < 0.05, indicates a significant difference between pre- and post-tests.

**Table 4:** Changes in time domain variables from pre- to post-tests.

		PRE	POST	Group	Time	G × T
R-R Intervals (n)	HIIT	304.7 ± 36.49	286.0 ± 49.06 ^+^	4.035	8.437 *	0.534
	MICT	348.3 ± 23.15	318 ± 30.48 ^+^			
IBI (sec)	HIIT	1.0 ± 0.11	1.1 ± 0.17 ^+^	4.575	9.611 *	0.032
	MICT	0.9 ± 0.06	0.9 ± 0.09 ^+^			
RMSSD	HIIT	0.1 ± 0.02	0.1 ± 0.02	2.057	0.214	1.536
	MICT	0.1 ± 0.04	0.1 ± 0.01			

IBI: inter-beat interval, RMSSD: root mean square of the successive differences. Values are expressed mean and standard deviations. * *p* < 0.05, indicates significant time or interaction effect, ^+^ *p* < 0.05, indicates a significant different between pre- and post-tests.
